# The Accuracy of 3D Printing Assistance in the Spinal Deformity Surgery

**DOI:** 10.1155/2019/7196528

**Published:** 2019-11-11

**Authors:** Po-Chen Chen, Chien-Chun Chang, Hsien-Te Chen, Chia-Yu Lin, Tsung-Yu Ho, Yen-Jen Chen, Chun-Hao Tsai, Hsi-Kai Tsou, Chih-Sheng Lin, Yi-Wen Chen, Horng-Chaung Hsu

**Affiliations:** ^1^Section of Orthopaedic Surgery, Department of Surgery, Ministry of Health and Welfare, Changhua Hospital, Changhua, Taiwan; ^2^Department of Orthopaedic Surgery, China Medical University Hospital, China Medical University, Taichung, Taiwan; ^3^Ph.D. Degree Program of Biomedical Science and Engineering, National Chiao Tung University, Hsinchu, Taiwan; ^4^Department of Sports Medicine, College of Health Care, China Medical University, Taichung, Taiwan; ^5^School of Medicine, China Medical University, Taichung, Taiwan; ^6^Functional Neurosurgery Division, Neurological Institute, Taichung Veterans General Hospital, Taichung, Taiwan; ^7^Department of Rehabilitation, Jen-Teh Junior College of Medicine, Nursing and Management, Miaoli County, Taiwan; ^8^Department of Biological Science and Technology, National Chiao Tung University, Hsinchu, Taiwan; ^9^Graduate Institute of Biomedical Sciences, China Medical University, Taichung, Taiwan; ^10^3D Printing Medical Research Institute, Asia University, Taichung, Taiwan

## Abstract

**Background:**

The pedicle screw is one of the main tools used in spinal deformity correction surgery. Robotic and navigated surgeries are usually used, and they provide superior accuracy in pedicle screw placement than free-hand and fluoroscopy-guided techniques. However, their high cost and space limitation are problematic. We provide a new solution using 3D printing technology to facilitate spinal deformity surgery.

**Methods:**

A workflow was developed to assist spinal deformity surgery using 3D printing technology. The trajectory and profile of pedicle screws were determined on the image system by the surgical team. The engineering team designed drill templates based on the bony surface anatomy and the trajectory of pedicle screws. Their effectiveness and safety were evaluated during a preoperative simulation surgery. The surgery consisted in making a pilot hole through the drill template on a computed tomography- (CT-) based, full-scale 3D spine model for every planned segment. Somatosensory evoke potential (SSEP) and motor evoke potential (MEP) were used for intraoperative neurophysiological monitoring. Postoperative CT was obtained 6 months after the correction surgery to confirm the screw accuracy.

**Results:**

From July 2015 to November 2016, we performed 10 spinal deformity surgeries with 3D printing technology assistance. In total, 173 pedicle screws were implanted using drill templates. No notable change in SSEP and MEP or neurologic deficit was noted. Based on postoperative CT scans, the acceptable rate was 97.1% (168/173). We recorded twelve pedicle screws with medial breach, six with lateral breach, and five with inferior breach. Medial breach (12/23) was the main type of penetration. Lateral breach occurred mostly in the concave side (5/6). Most penetrations occurred above the T8 level (69.6%, 16/23).

**Conclusion:**

3D printing technology provides an effective alternative for spinal deformity surgery when expensive medical equipment, such as intraoperative navigation and robotic systems, is unavailable.

## 1. Introduction

The pedicle screw is one of the most important tools in spinal deformity correction surgery [1]. However, the abnormal anatomy and pedicle morphology increase the difficulty in applying pedicle screws. The rotational deformities also make it more difficult to set the appropriate angle of pedicle screw and to determine the pedicle breach using fluoroscopy intraoperatively [2, 3]. A systematic review in 2012 reported that in studies using free-hand technique, the accuracy ranged from 69% to 94%; with the aid of fluoroscopy, it ranged from 28% to 85% [4]. In addition to inaccuracy, the wide range of variations in free-hand and fluoroscopy-guided techniques may affect the outcomes. Navigation and robot-guided surgery could be used to mitigate inaccuracy and high variation [4–6]. However, the high acquisition and maintenance costs of such equipment, as well as the spatial limitation of operating rooms and the need of multiple scans in long-segment surgery, limit the use of these techniques [5].

We developed a technique to overcome the limitations of the aforementioned techniques. The technique employs 3D printing technology to assist in spinal deformity surgery and applies drill templates to increase the accuracy of pedicle screw placement. The causes of inaccurate placement with this technique were also analysed.

## 2. Materials and Methods

### 2.1. Manufacture of a Spine Model and a Drill Template

**Figure 1:**
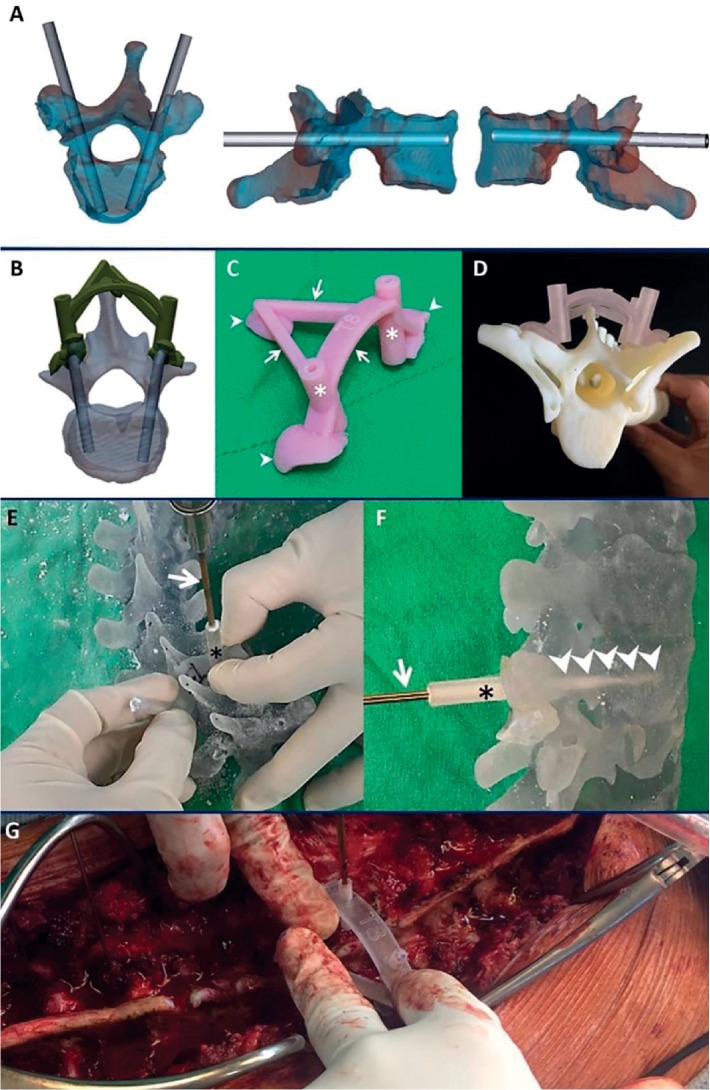
(a) The trajectory, diameter, and length of the pedicle screws are planned on the Avizo software. (b) The drill template is designed on Geomagic Design X software based on the profiles of pedicle screw and the anatomic traits of a certain level. (c) The drill template is composed of three parts, namely, the foot template (arrowhead), drilling cannula (asterisk), and connecting bar (arrow). (d) Finished products of 3D printing technology in this study, including the drill template and the 3D spine model. (e and f) The surgeon uses a power drill (arrow) to create a pilot hole on the full-scale spine model during the simulation surgery. The trajectory is guided by the drilling cannula (asterisk) of the drill template, and the route can be directly visible (arrowhead). (g) The drill template is mounted on the patient's spine.

To test the validity of 3D printing technology in treating spinal deformity, we designed a treatment protocol. Scoliosis patients who required surgical correction were enrolled. All patients received a whole spine CT (0.625-mm-thick slice) before surgery, and the Avizo software (FEI, Burlington, MA, USA) was used to generate a 3D model. A full-scale spine model was created using a 3D printer (Stratasys Connex3 Objet500) with some VeroClear (Stratasys, Eden Prairie, MN, USA) material. The trajectory, diameter, and length of the pedicle screws were planned on the image system Avizo software (Figure 1(a)), and the drill template was designed on Geomagic Design X software based on the profiles of pedicle screw and the anatomic traits of certain levels (Figure 1(b)). The drill template was created using a 3D printer (Stratasys Objet30 OrthoDesk) with MED610 material. The drill template comprised three parts, namely, a foot template, drilling cannula, and connecting bar (Figure 1(c)). The patient-specific foot template can maintain close contact to the posterior surface of certain level. Three foot templates were designed to increase stability during the application of the drill. The connecting bar was to strengthen the rigidity of the drill template. The drilling cannula was a hallowed column with a 4.5 mm outer diameter and a 2.5 mm inner diameter, allowing a 2.5 mm drill bit to pass through.

### 2.2. Preoperative Simulation Surgery

A simulation surgery was performed before the formal operation. Pilot holes were drilled on a full-scale spine model with the drill templates. All routes were directly visible (Figures 1(e) and 1(f)) and were checked to verify their suitability. If necessary, the design of certain levels could be revised and retested. The whole spine model and drill templates were recreated and sterilised using an ethylene oxide sterilisation process after passing the examination.

### 2.3. Operation Procedure

**Figure 2:**
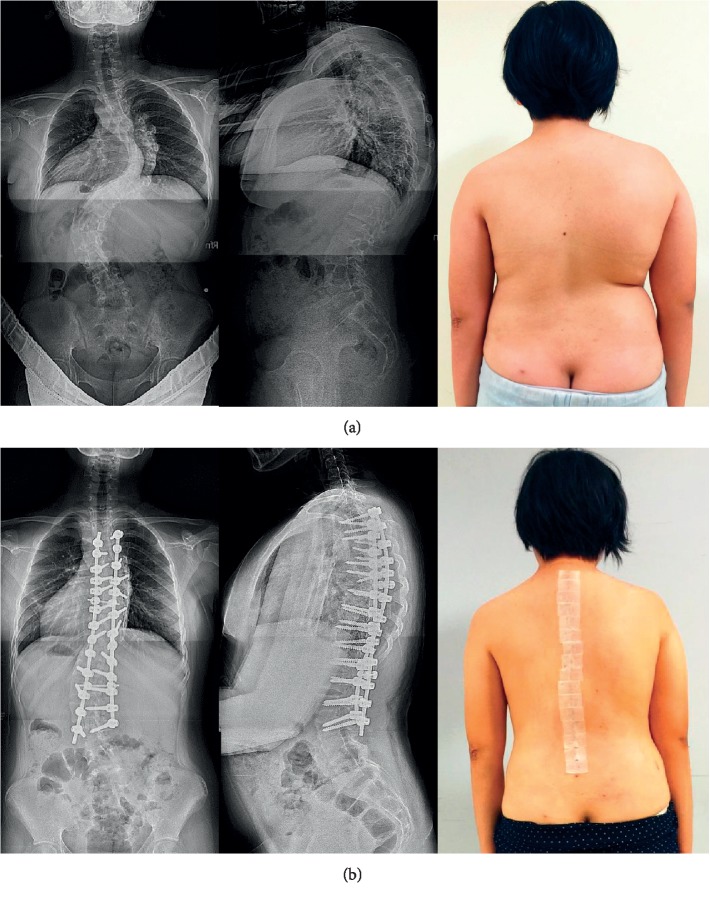
(a) Anteroposterior and lateral scanography of spine and photograph before surgery (Case 1). (b) Anteroposterior and lateral scanography of spine and photograph after surgery (case 1).

Patient-specific full-scale spine models were available as a reference during the operation. All patients received intraoperative neurophysiological monitoring, including somatosensory evoke potential (SSEP) and motor evoke potential (MEP). After all pilot holes were drilled using the drill templates, guide pins were inserted into the pilot holes and fluoroscopy confirmation was performed. Cannulated pedicle screws (Medtronic Longitude) were then positioned. All surgeries were performed by a spinal surgeon. A typical case is shown in Figure 2.

### 2.4. Data Collection and Analysis

**Figure 3:**
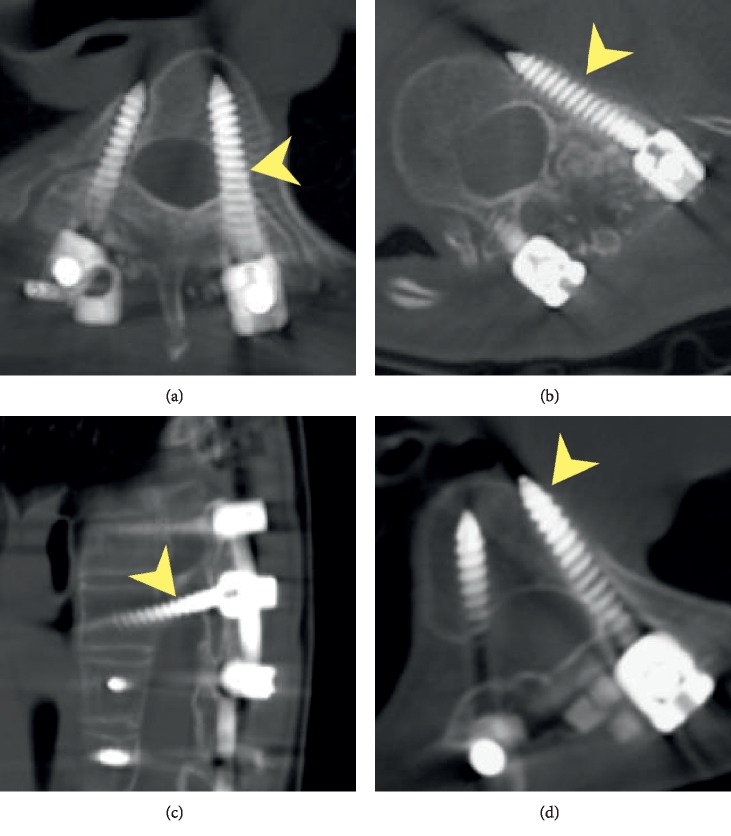
(a) Medial breach (arrowhead) is defined as violation of medial pedicle wall. (Case 10, T5). (b) Lateral breach (arrowhead) is defined as violation of lateral pedicle wall. (Case 10, T12). (c) Inferior breach (arrowhead) is defined as violation of inferior pedicle wall. (Case 2, T7). (d) Anterior breach (arrowhead) is defined as penetration of vertebral body without pedicle wall violation (not in this case series).

Neurological symptoms and operation-related complications were assessed and recorded by a member of the surgical team. All patients received a whole spine CT 6 months after the surgery, and the CT scans were assessed by an orthopaedic surgeon outside the surgical team. The accuracy was defined according to the proportion of pedicle screws without any breach, and the acceptability was defined based on the proportion of pedicle screws with less than 2 mm breaches. Types of screw breach were categorized based on the direction of misplacement (Figure 3). Statistical analyses were performed using SPSS. All *p* values were calculated through permutation tests.

## 3. Results

Between July 28, 2015, and November 29, 2016, 10 patients were treated using this protocol: six patients with adolescent idiopathic scoliosis (AIS), three patients with congenital scoliosis, and one patient with neuromuscular scoliosis. A total of 173 pedicle screws were placed using drill templates. No significant SSEP or MEP change was recorded intraoperatively, and no neurological symptoms were noted postoperatively. One patient had pneumothorax due to the correction of the deformity. No notable lateral breach or anterior breach was recorded on this patient (Tables 1 and 2).

The accuracy ranged from 61.1% to 100% (mean: 86.7%, 150 out of 173 screws). The acceptability ranged from 88.4% to 100% (mean: 97.1%, 168 out of 173 screws). Most screw breaches were within 2 mm (Grade A, 18/23). Medial breach was the most common type (12/23) (Table 3).

The risk factors of pedicle screw breach were analysed. Pedicle screw placement above the T8 level had a significantly higher risk of breach than that below the T9 level (above T8: 17 screw breaches out of 59 screws; below T9: 6 screw breaches out of 114 screws. *p* value = 0.0001). In our study, lateral breach more often occurred on the concave side; inferior breach more often occurred on the convex side. However, there was no statistically significant difference (Table 4).

## 4. Discussion

Based on our review, the 3D printed drill template provides higher accuracy, acceptability, and less variation than the free-hand technique and fluoroscopy-assisted method [4, 7] (Table 5). It is comparable to the navigation method, without its high cost and spatial limitation. Yang et al. [8] compared those who use the 3D printing spine model for preoperative planning with those who did not. The spine model group showed less operation time and blood loss; meanwhile, the hospital cost was 4500 Ren Min Bi (RMB) higher than the control group in average. A systematic review in 2017 [9] concluded that the 3D model would be of extra cost between $300 and >$1,000, and it also revealed that 3D printing guides can offer a simple, convenient, low cost, and complex equipment-free way to improve the accuracy of pedicle screw placement. The manufacture of spine model and drill template can also be outsourced, like the applications of patient-specific instrumentation (PSI) in knee replacement. A randomized controlled trial [10] proved that PSI and single use instrumentation (SUI) is a cost-saving method for healthcare providers.

### 4.1. Causes of Inaccuracy

According to our study, the major risk factor of inaccuracy is screw placement above the level of T8. The precision of the drill template relies on the extent of fitness to the bone surface [11, 12]. Thus, a higher-level spine with a smaller pedicle had less tolerance to soft tissue interposition between the foot process and bone surface [2, 13]. Thorough excision of the soft tissue beneath the foot template is crucial. Some of the pedicles in the concave side were even too narrow to accommodate the inserted screw. In addition, a smaller pedicle consists of a higher proportion of cortical bone [2, 14, 15]. A slow advance of the drill while maintaining high revolutions per minute is another method to prevent trajectory deviation.

O'Brien et al. [2] reported that rotational deformity, rather than a smaller pedicle size, compromises the accuracy of the pedicle screw placement in scoliotic spines, especially on the concave side. However, the data from our results did not support this assumption (Table 5). We observed that the use of a 3D model and drill templates partially mitigated the difficulty of pedicle screw placement caused by rotational deformity. Previous studies have also revealed that the use of a 3D model could minimise the surgical time, blood loss, and need for blood transfusion, as well as increasing the accuracy [8, 16, 17].

The entry point of a thoracic pedicle screw is usually on the slope of the transverse process [18, 19]. The drill tip may slide medially on the hard cortex surface while drilling, which may cause the medial shift of the entry point and increase the convergence, thus resulting in medial breach. The solution is to prepare the entry point by using a high-speed burr, thus exposing the cancellous bone and making the slope more even. These recommendations enable the achievement of the planned trajectory.

During the dissection of the concave side, the surgical field is deeper and more limited, and the remaining soft tissue may interpose between the drill template and the bone surface causing lateral breach [20]. This condition can be prevented by a thorough excision of soft tissue beneath the foot template of the drill template. While applying the pedicle screw, the trajectory may be interfered by rib and overlying tissue blockage, causing a decrease in the convergence and resulting in lateral breach, especially on the concave side of a torsional spine. Surgeons must maintain the original convergence when applying the pedicle screw, and they should remove the blocking rib and tissue accordingly.

According to our study, medial breach was the most common type of screw violation; lateral breach was more likely to occur on the concave side; and inferior breach was more common on the convex side. Nevertheless, these results were not statistically significant. Further studies based on a larger sample size and control group are necessary.

**Figure 4:**
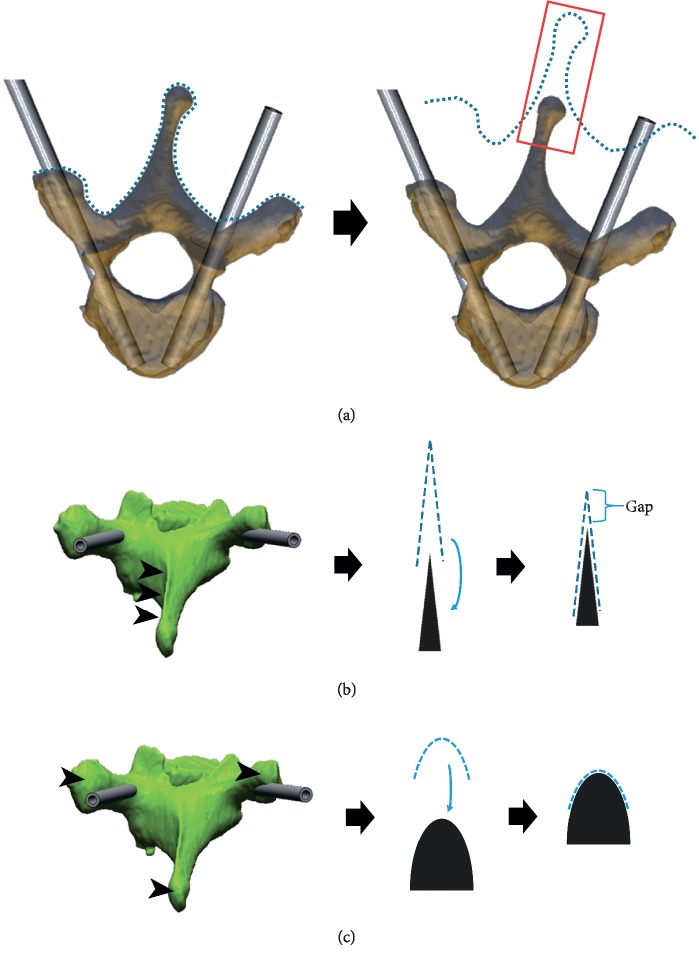
(a) The drill template is the female die (dotted line) on the target zone of the patient's spine. In practice, the target zone should be carefully selected to avoid inapplicability (box). (b) The sharply curved template (dotted line) generates high friction between the template and the target zone (arrowhead), resulting in a gap that causes inaccuracy. (c) The smoothly curved template (dotted line) creates a close fit between the template and the target zone (arrowhead), insuring the planned trajectory.

In addition to the technical errors encountered using the drill templates, the following factors caused inaccuracy: improper design, discontinuity of the CT scan, inaccuracy of the 3D image generation, and error from the 3D printing. In the case of improper design, for example, several minor errors may result in a substantial error when using multisegment drill templates. If the foot template is developed based on the facet joints or anatomies of different segments, the accuracy may be influenced when the patient is in different positions [7, 11, 12, 20]. Some anatomies were not suitable for the manufacture of drill templates because of their inapplicability (Figure 4(a)). Because foot templates are the female die of the surface anatomy, they have a rough surface. Friction was observed when applying them. When a foot template is designed based on a spinous process, the angle is too sharp to apply the template (Figures 4(b) and 4(c)). Applying such template also requires removing the supraspinous and interspinous ligaments, and thus, it compromises the stability. A 3D image was generated from discontinuous CT images. Thinner CT slices can minimise the distortion. Errors might also occur during the 3D printing, which is difficult to prevent. Therefore, a simulation surgery before the real surgery is essential.

### 4.2. Design Rationale

These 3D printing templates can guide the orientation and depth not only in drilling but also in the cutting and burring process [21]. Lu et al. [20], Feng et al. [22],and Chen et al. [23] designed their template's undersurface as the inverse of spinous process, transverse process, and lamina, enabling a fit in a lock-and-key fashion. We have tried this fashion before and in our experience, it may improve the stability. However, the difficulty in applying the template would be a concern. The surgeon may have to press hard for the template to fit in, and sometimes, the thin template would break due to its limited elasticity. Sugawara et al. [24] designed their template in a spinous-process-free fashion. This type is easier to apply in our experience. Besides, their design consisted of three types of template, guiding the entry point marking, pilot hole drilling, and screw placement. Despite this, the extensive soft tissue dissection is still crucial to this design. Drill templates with a larger contact area can theoretically ensure higher precision, but they also require thorough excision of the soft tissue, which causes more damage and postpones the patient's recovery. Chen et al. [13] used a 2-foot-template design to minimise the dissection. However, the two-point-contact design is less stable than the three-point-contact structure, and the short drilling cannula may compromise the accuracy of drilling. Berry et al. [11] used drill templates with three support points and a handle to stabilise the template. Therefore, their design was relatively thick and bulky. Furthermore, Berry et al. [11] used DuraForm polyamide (3D Systems, Andover, MA, USA) as the drill template material, which does not deform after autoclaving. Our design consists of three small-contact-area foot templates, which are stabilised by three connecting bars. This design minimises the need of soft tissue dissection without compromising stability or accuracy. The low weight of the design and its transparent material also facilitate direct visual observation while drilling, enabling the surgeon to see whether the entry point is proper.

## 5. Conclusion

The use of 3D printing technology to assist spinal deformity surgery with drill templates provides high accuracy, acceptability, and consistency. It is a viable alternative method to navigation surgery.

## Figures and Tables

**Table 1:** Details of patients who underwent surgery.

No.	Age/gender	Diagnosis	Follow-up (months)	No. of screws	Accuracy	Acceptability
1^*∗*^	13/F	AIS, Lenke type 3C, T3-L3	40	26	80.8% (22/26)	100% (26/26)
2	13/F	AIS, Lenke type 1, T4-T12	39	18	61.1% (11/18)	94% (17/18)
3	13/F	Congenital scoliosis, L1-L4	34	8	100% (8/8)	100% (8/8)
4	7/M	Congenital scoliosis, L3-L5	33	5	100% (5/5)	100% (5/5)
5	15/F	AIS, Lenke type 3C, T4-L2	29	20	95% (19/20)	100% (20/20)
6	14/M	Congenital scoliosis, T8-L2	28	12	100% (12/12)	100% (12/12)
7	18/F	AIS, Lenke type 1BN, T4-L1	28	19	94.7% (18/19)	94.7% (18/19)
8	28/F	AIS, Lenke type 5, T6-L3	25	17	88.2% (15/17)	100% (17/17)
9	14/F	AIS, Lenke type 6, T5-L3	25	22	100% (22/22)	100% (22/22)
10	54/M	Neuromuscular scoliosis, T3-S2	24	26	69.2% (18/26)	88.5% (23/26)

^*∗*^This patient had pneumothorax due to the correction of the deformity. AIS = adolescent idiopathic scoliosis.

**Table 2:** Details of instrumentation level, type, grading, and side of pedicle screw breach.

No.	T3	T4	T5	T6	T7	T8	T9	T10	T11	T12	L1	L2	L3	L4	L5	S1	Total
1	MA^c^	MA^c^		LA^v^	LA^v^												2MA, 2LA
2		IA^v^LA^c^	MA^c^IA^v^		IB^v^	IA^v^				MA^v^							2MA, 1LA, 3IA, 1IB
3																	
4																	
5		IA^v^															1IA
6																	
7						MB^v^											1MB
8												MA^v^	MA^c^				2MA
9																	
10		MA^c^	MB^v^LA^c^	MB^c^		MA^v^		LA^v^		LC^c^					MA^c^		3MA, 2MB, 2LA, 1LC

Breach type: M: medial breach; L: lateral breach; I: inferior breach. Breach grading: A: <2 mm breach; B: ≥2 mm breach and <4 mm breach; C: ≥4 mm breach.^c^Concave side.^v^Convex side. Grey background: range of instrumentation.

**Table 3:** Number, type, and grading of pedicle screw breach.

	Medial breach	Lateral breach	Inferior breach	Anterior breach
Grade A	9	5	4	0
Grade B	3	0	1	0
Grade C	0	1	0	0
Total	12	6	5	0

Grade C: ≥4-mm breach. Grade A: <2-mm breach. Grade B: ≥2-mm breach and <4-mm breach.

**Table 4:** Number of penetration on the convex and concave sides.

	Convex	Concave	*p* value
Penetration	13/88	10/85	0.6563
Medial breach	7	5	0.9547
Lateral breach	1	5	0.2796
Inferior breach	5	0	0.1181

**Table 5:** Comparison of accuracy and acceptability among studies.

Study	Technique	Accuracy		Acceptability	
		Range	Mean	Range	Mean
Our study	Drill template	61.1%–100%	86.7%	88.5%–100%	97.1%
Liu et al.	Drill template	NA	93.8%	100%	100%
	Free hand	NA	78.8%	NA	97.1%
Gelalis et al.^*∗*^	Free hand	68.6%–94.2%	78.8%	76.3%–97.0%	89.0%
	2D fluoroscopy	27.6%–81.7%	58.1%	71.3%–95.2%	85.4%

NA = not available. ^*∗*^Studies without details of breach grading were excluded from calculation of acceptability.

## Data Availability

The data are available on request from the author Po-Chen Chen via e-mail: ftsystar@gmail.com.

## Abbreviations

3D: Three-dimensional
CT: Computed tomography
SSEP: Somatosensory evoke potential
MEP: Motor evoke potential.

## References

[1] Ledonio C. G. T., Polly D. W., Vitale M. G., Wang Q., Richards B. S. (2011). Pediatric pedicle screws: comparative effectiveness and safety. *The Journal of Bone and Joint Surgery-American Volume*.

[2] O’Brien M. F., Lenke L. G., Mardjetko S. (2000). Pedicle morphology in thoracic adolescent idiopathic scoliosis: is pedicle fixation an anatomically viable technique?. *Spine*.

[3] Kim Y. J., Lenke L. G., Cheh G., Riew K. D. (2005). Evaluation of pedicle screw placement in the deformed spine using intraoperative plain radiographs: a comparison with computerized tomography. *Spine*.

[4] Gelalis I. D., Paschos N. K., Pakos E. E. (2012). Accuracy of pedicle screw placement: a systematic review of prospective in vivo studies comparing free hand, fluoroscopy guidance and navigation techniques. *European Spine Journal*.

[5] Staartjes V. E., Klukowska A. M., Schröder M. L. (2018). Pedicle screw revision in robot-guided, navigated, and freehand thoracolumbar instrumentation: a systematic review and meta-analysis. *World Neurosurgery*.

[6] Fan Y., Du J. P., Liu J. J. (2018). Accuracy of pedicle screw placement comparing robot-assisted technology and the free-hand with fluoroscopy-guided method in spine surgery: an updated meta-analysis. *Medicine*.

[7] Liu K., Zhang Q., Li X. (2017). Preliminary application of a multi-level 3D printing drill guide template for pedicle screw placement in severe and rigid scoliosis. *European Spine Journal*.

[8] Yang M., Li C., Li Y. (2015). Application of 3D rapid prototyping technology in posterior corrective surgery for Lenke 1 adolescent idiopathic scoliosis patients. *Medicine*.

[9] Wilcox B., Mobbs R. J., Wu A.-M., Phan K. (2017). Systematic review of 3D printing in spinal surgery: the current state of play. *Journal of Spine Surgery*.

[10] Attard A., Tawy G. F., Simons M., Riches P., Rowe P., Biant L. C. (2019). Health costs and efficiencies of patient-specific and single-use instrumentation in total knee arthroplasty: a randomised controlled trial. *BMJ Open Quality*.

[11] Berry E., Cuppone M., Porada S. (2005). Personalised image-based templates for intra-operative guidance. *Proceedings of the Institution of Mechanical Engineers, Part H: Journal of Engineering in Medicine*.

[12] Lu S., Xu Y. Q., Zhang Y. Z. (2009). A novel computer-assisted drill guide template for lumbar pedicle screw placement: a cadaveric and clinical study. *The International Journal of Medical Robotics and Computer Assisted Surgery*.

[13] Chen H., Guo K., Yang H., Wu D., Yuan F. (2016). Thoracic pedicle screw placement guide plate produced by three-dimensional (3-D) laser printing. *Medical Science Monitor*.

[14] Abul-Kasim K., Ohlin A. (2012). Patients with adolescent idiopathic scoliosis of Lenke type-1 curve exhibit specific pedicle width pattern. *European Spine Journal*.

[15] Davis C. M., Grant C. A., Pearcy M. J. (2017). Is there asymmetry between the concave and convex pedicles in adolescent idiopathic scoliosis? A CT investigation. *Clinical Orthopaedics and Related Research*.

[16] Guarino J., Tennyson S., McCain G., Bond L., Shea K., King H. (2007). Rapid prototyping technology for surgeries of the pediatric spine and pelvis. *Journal of Pediatric Orthopaedics*.

[17] Provaggi E., Leong J. J. H., Kalaskar D. M. (2017). Applications of 3D printing in the management of severe spinal conditions. *Proceedings of the Institution of Mechanical Engineers, Part H: Journal of Engineering in Medicine*.

[18] Lehman R. A., Polly D. W., Kuklo T. R., Cunningham B., Kirk K. L., Belmont P. J. (2003). Straight-forward versus anatomic trajectory technique of thoracic pedicle screw fixation: a biomechanical analysis. *Spine*.

[19] Fennell V. S., Palejwala S., Skoch J., Stidd D. A., Baaj A. A. (2014). Freehand thoracic pedicle screw technique using a uniform entry point and sagittal trajectory for all levels: preliminary clinical experience. *Journal of Neurosurgery: Spine*.

[20] Lu S., Zhang Y. Z., Wang Z. (2012). Accuracy and efficacy of thoracic pedicle screws in scoliosis with patient-specific drill template. *Medical & Biological Engineering & Computing*.

[21] Rong X., Wang B., Chen H. (2016). Use of rapid prototyping drill template for the expansive open door laminoplasty: a cadaveric study. *Clinical Neurology and Neurosurgery*.

[22] Feng Z.-H., Li X.-B., Phan K. (2018). Design of a 3D navigation template to guide the screw trajectory in spine: a step-by-step approach using mimics and 3-matic software. *Journal of Spine Surgery*.

[23] Chen X. L., Xie Y. F., Li J. X. (2019). Design and basic research on accuracy of a novel individualized three-dimensional printed navigation template in atlantoaxial pedicle screw placement. *PLoS One*.

[24] Sugawara T., Kaneyama S., Higashiyama N. (2018). Prospective multicenter study of a multistep screw insertion technique using patient-specific screw guide templates for the cervical and thoracic spine. *Spine*.

